# Supplementary material for: Integrative genomic analyses of promoter G-quadruplexes reveal their selective constraint and association with gene activation
Source: Commun Biol. 2023 Jun 10;6:625. doi: 10.1038/s42003-023-05015-6 (PMC10257653; doi:10.1038/s42003-023-05015-6)
Supplement: Supplementary file 2 — Supplementary Information [file 42003_2023_5015_MOESM2_ESM.pdf]

Supplementary Information for

Integrative genomic analyses of promoter G-quadruplexes reveal their selective constraint and association with gene activation

Guangyue Li[1], Gongbo Su[1], Yunxuan Wang[2], Wenmeng Wang[1], Jinming Shi[1], Dangdang Li[1] and Guangchao Sui[1,*]

[1]College of Life Science, Northeast Forestry University, Harbin 150040, China.

[2]Department of Medical Oncology, Harbin Medical University Cancer Hospital, Harbin, 150081, China

* To whom correspondence should be addressed. Tel: +86-451-82191081; E-mail: gcsui@nefu.edu.cn

This PDF file includes Supplementary Figure 1-9 with legends.

Supplementary Figures

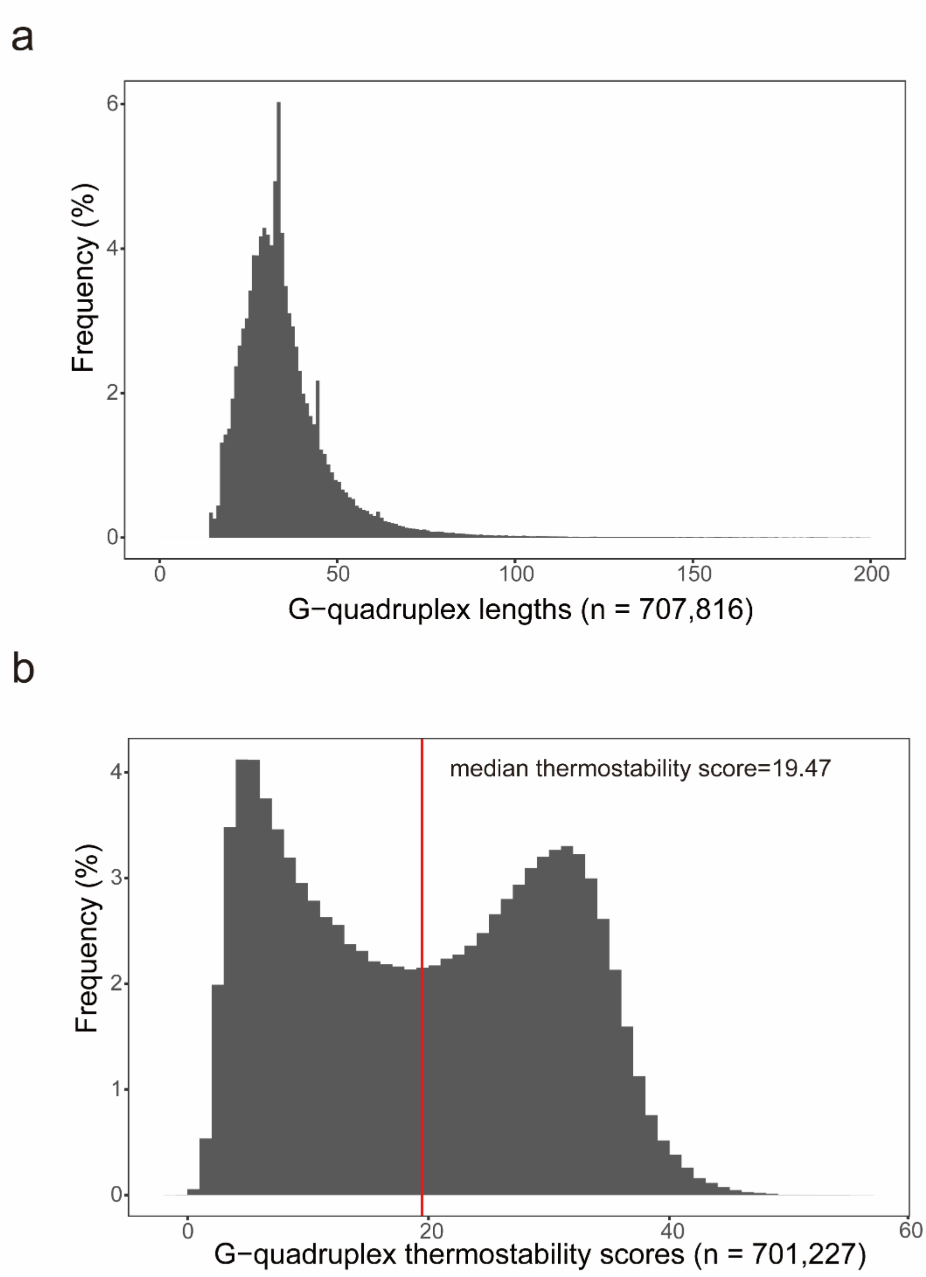

Supplementary Figure 1. Distributions of the lengths (**a**) and thermal stability scores (**b**) of putative G4s (pG4s) in the human genome. The scores of pG4 thermal stability were calculated using determined by Quadron software[1].

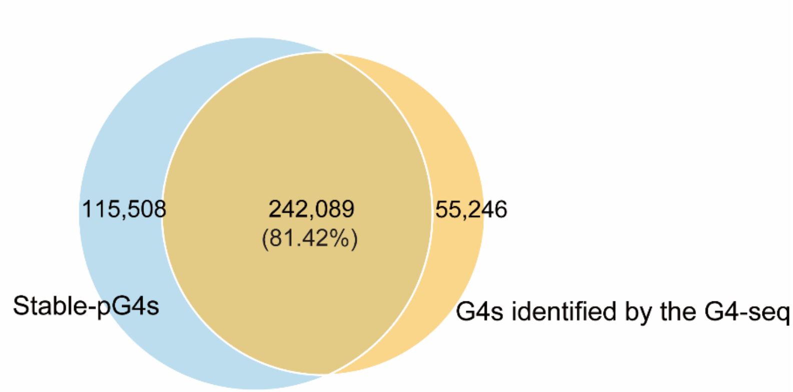

Supplementary Figure 2. Venn plot of stable-pG4s with thermal stability scores greater than 19 determined by Quadron software and G4s identified by the G4-seq[2].

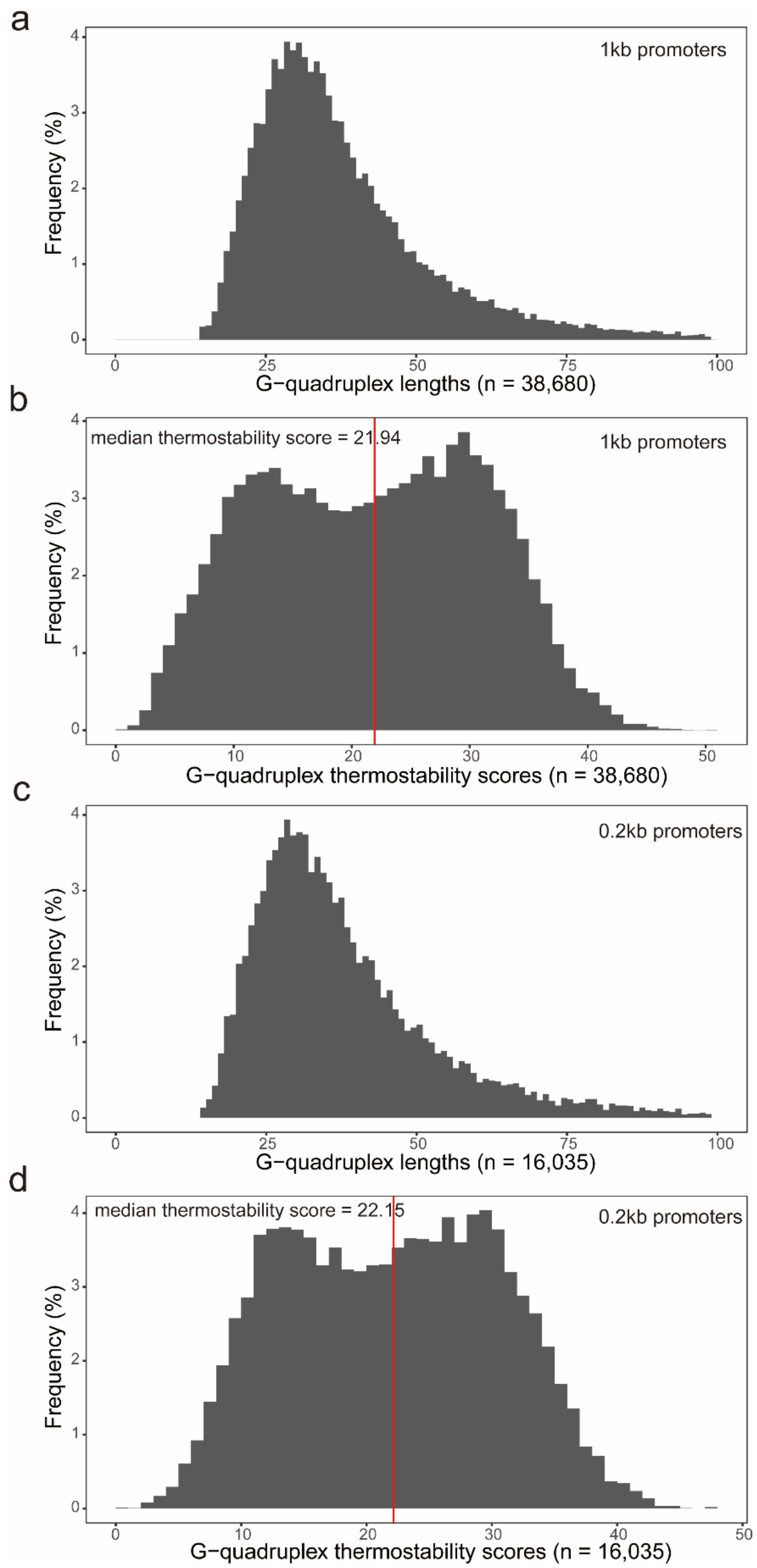

Supplementary Figure 3. Distribution of the lengths and thermal stability scores of pG4s in 1kb promoters (**a** and **b**, respectively) and 0.2kb promoters (**c** and **d**, respectively). The red lines represent the median values of the thermos stability scores in 1kb and 0.2kb promoters.

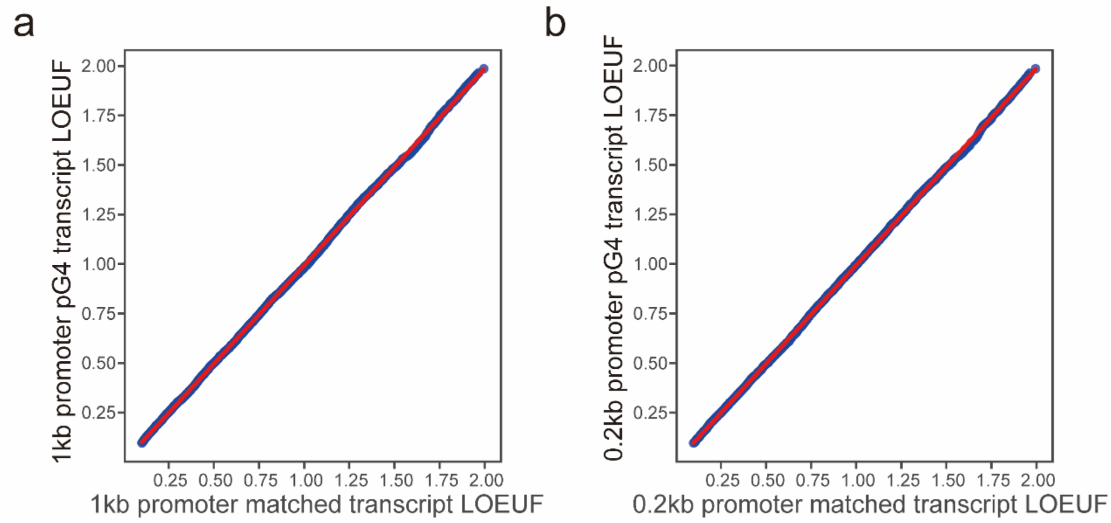

Supplementary Figure 4. Quantile-quantile plots showing the consistency of LOEUF score distributions of pG4 and non-pG4 transcripts in 1kb (**a**) and 0.2kb (**b**) promoters. Allele frequencies (Figure 1f, g and supplementary Figure 5) were compared in constraint-matched transcripts using LOEUF metric[3] to control for the possibility that nearby constrained coding sequences might affect local allele frequency estimates.

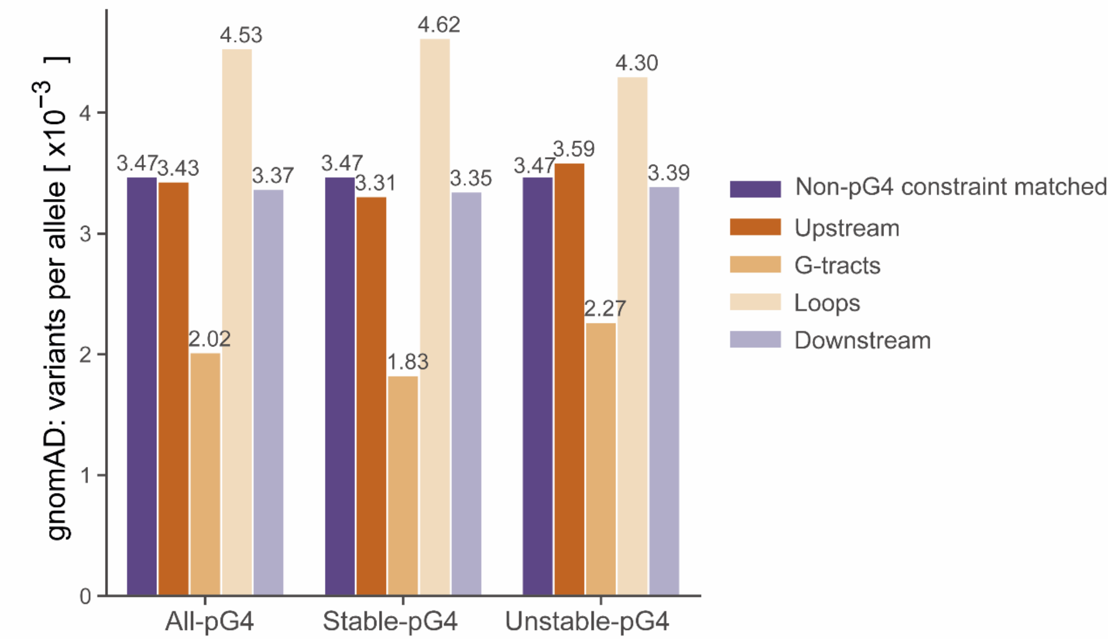

Supplementary Figure 5. Allele frequencies of pG4s in 0.2k promoters. Allele frequencies in G-tracts, loops, 100 bp regions upstream and downstream of pG4s, and LOEUF-constrained non-PG4 regions.

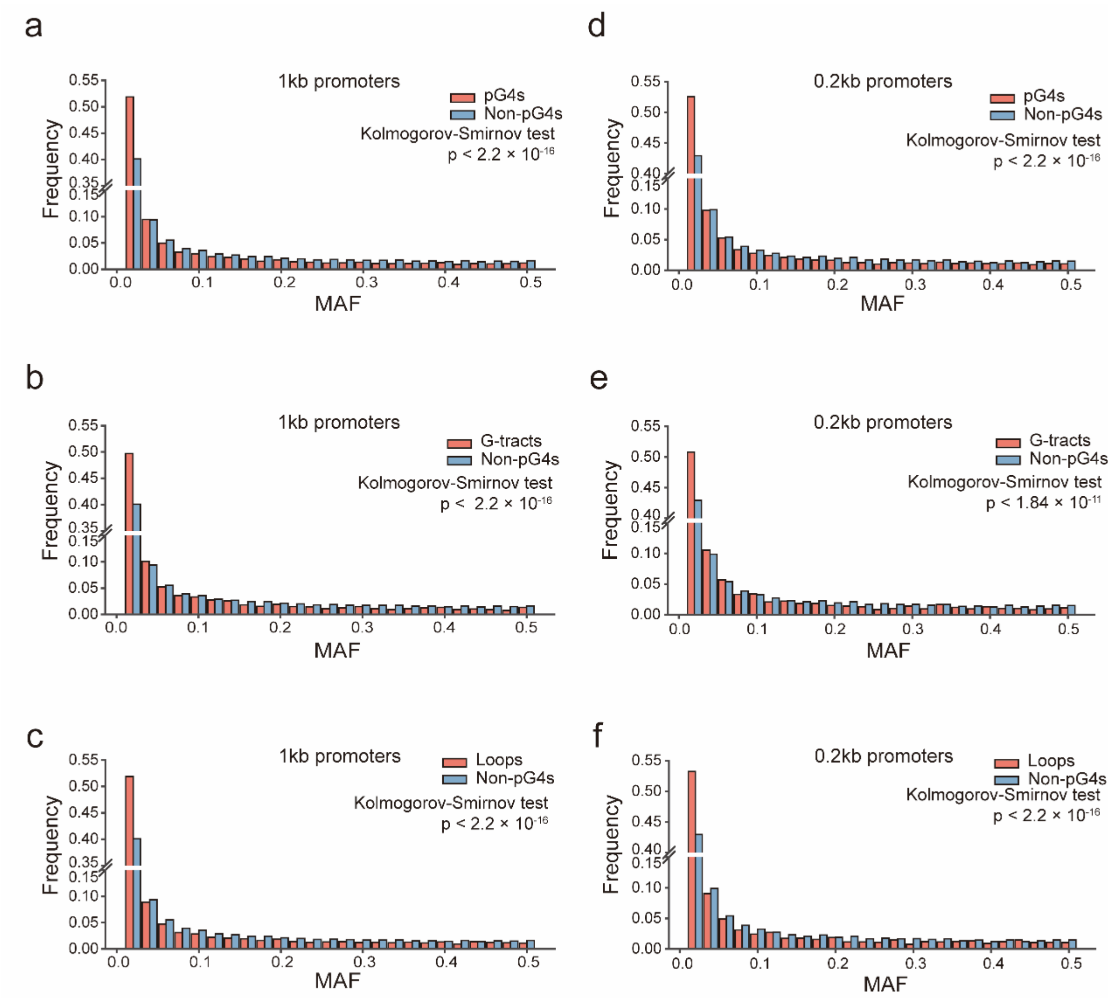

Supplementary Figure 6. Allele frequency spectra of pG4s, pG4 G-tracts and loops. Histograms of Minor Allele Frequencies (MAFs) of SNPs from the population of the EUR of 1000 Genomes project[4] discovered in 1kb promoters (a-c) and 0.2kb promoter (d-f). SNPs are split into intersecting G4 loci or not (non-pG4s). Comparisons between the MAF distribution were performed with the two-sample Kolmogorov–Smirnov test. Singletons and doubletons were excluded.

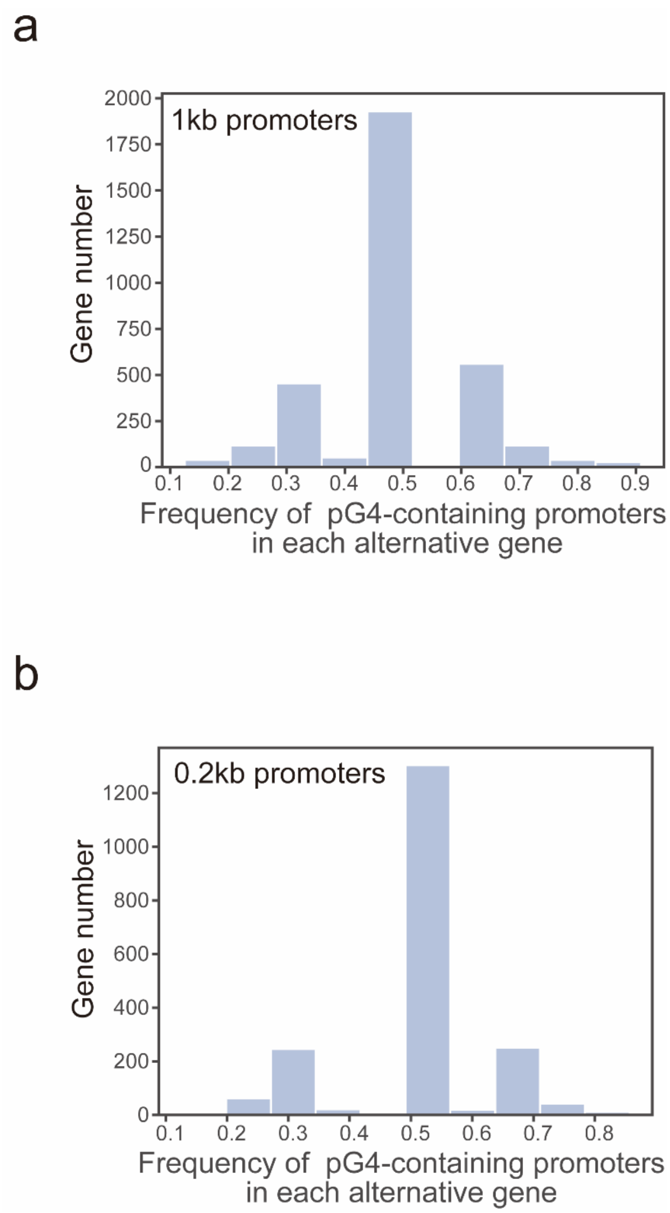

Supplementary Figure 7. The distribution of the ratios of pG4-containing 1kb promoters (**a**) or 0.2kb promoters (**b**) versus all promoters in alternative genes.

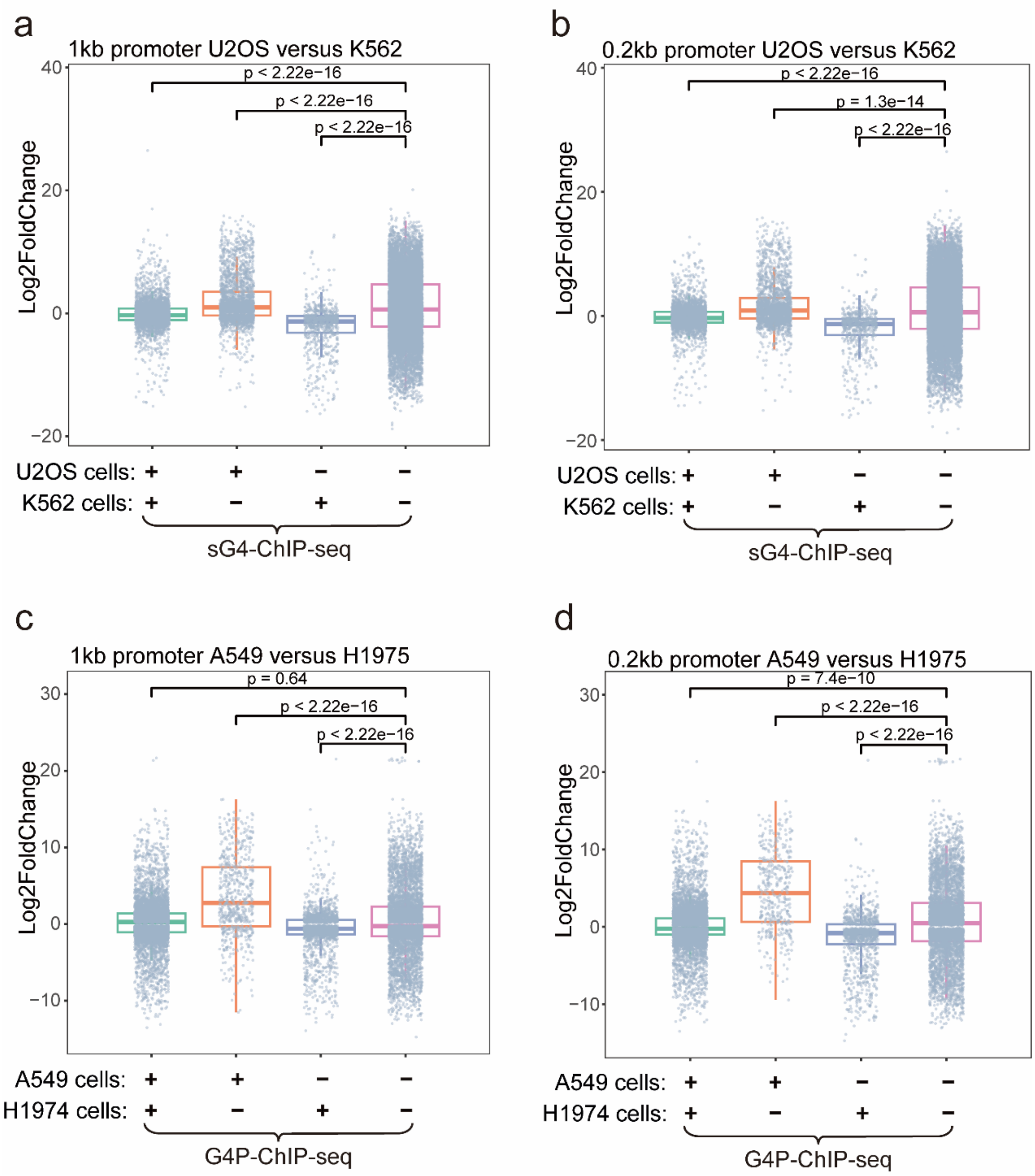

Supplementary Figure 8. Comparison of individual 1kb (**a** and **c**) and 0.2kb (**b** and **d**) promoter activities of U2OS[5] versus K562[6] (**a** and **b**) cell lines and A549[7] versus H1975[8] (**c** and **d**) cell lines based on RNA-seq data. The promoters are put into 4 groups according to their G4 structure statuses determined by G4-ChIP-seq analyses (sG4-ChIP-seq[9], G4P-ChIP-seq[10]). Each box plot (central line, median, box limits, upper and lower quartiles) represents the log2-fold-change on each group.

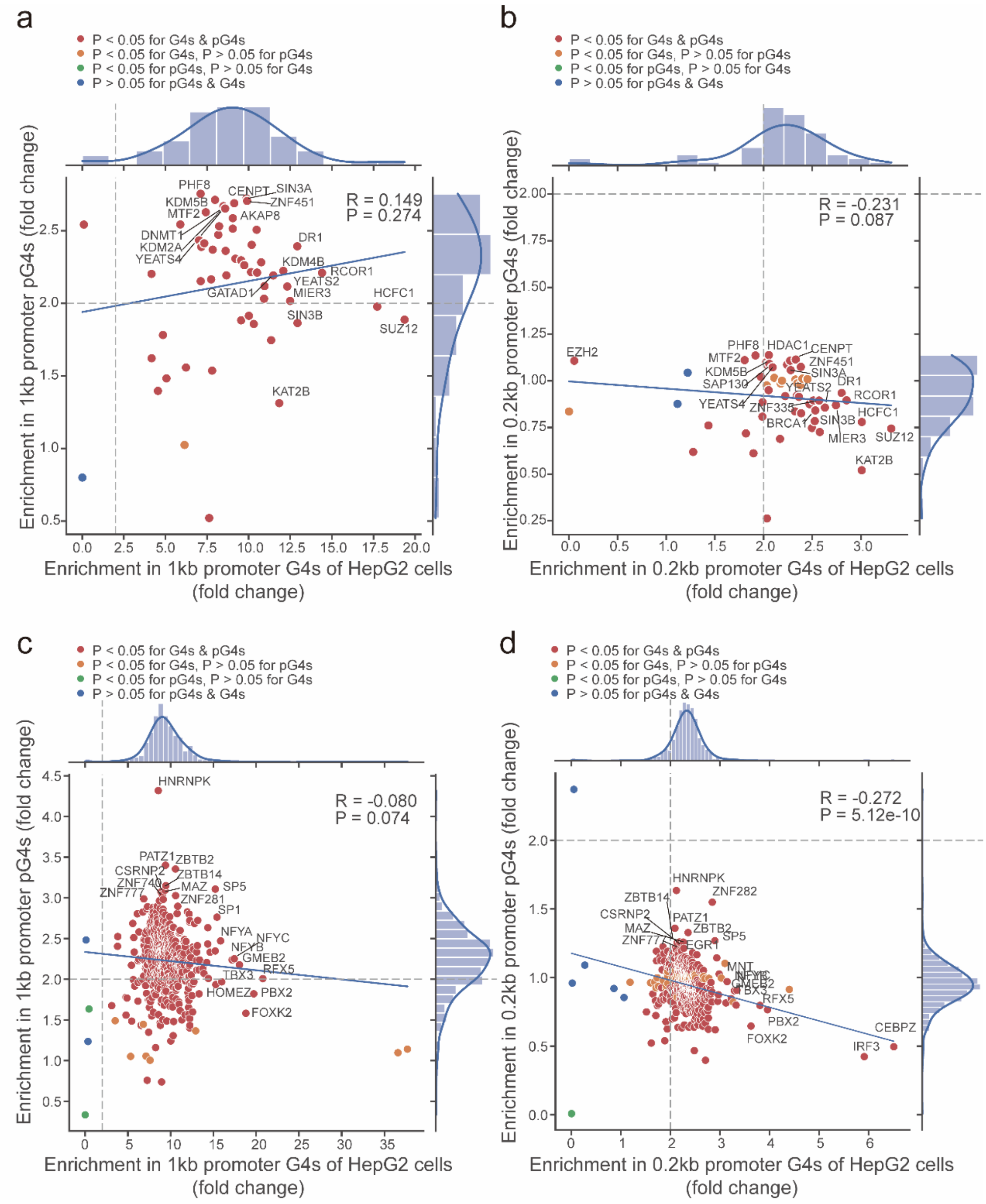

Supplementary Figure 9. Correlation of pG4s and experimentally identified G4s with chromatin remodeling proteins and TFs in promoters in HepG2 cells.

(**a**)-(**d**) Scatter plots of pG4s and BG4-ChIP-seq-identified G4s versus 56 chromatin remodeling proteins (**a** and **b**) and 505 TFs (**c** and **d**) in 1kb promoters (**a** and **c**) and 0.2kb promoters (**b** and **d**) in HepG2 cells based on the ChIP-seq data obtained from the ENCODE database. Correlations with $P < 0.05$ and $P > 0.05$ are presented by different color dots as indicated. The overall correlation coefficient and P value of each

graph are provided.

Supplementary References

1. Sahakyan AB, Chambers VS, Marsico G, Santner T, Di Antonio M, Balasubramanian S. Machine learning model for sequence-driven DNA G-quadruplex formation. *Sci Rep* **7**, 14535 (2017).
2. Chambers VS, Marsico G, Boutell JM, Di Antonio M, Smith GP, Balasubramanian S. High-throughput sequencing of DNA G-quadruplex structures in the human genome. *Nat Biotechnol* **33**, 877-881 (2015).
3. Karczewski KJ*, et al.* The mutational constraint spectrum quantified from variation in 141,456 humans. *Nature* **581**, 434-443 (2020).
4. Fairley S, Lowy-Gallego E, Perry E, Flicek P. The International Genome Sample Resource (IGSR) collection of open human genomic variation resources. *Nucleic Acids Res* **48**, D941-D947 (2020).
5. Schwab K*, et al.* Multi-omics analysis identifies RFX7 targets involved in tumor suppression and neuronal processes. *Cell Death Discov* **9**, 80 (2023).
6. Li X*, et al.* Chromatin context-dependent regulation and epigenetic manipulation of prime editing. *bioRxiv*, (2023).
7. Feng J*, et al.* Targeting metabolic vulnerability in mitochondria conquers MEK inhibitor resistance in KRAS-mutant lung cancer. *Acta Pharm Sin B* **13**, 1145-1163 (2023).
8. Gogleva A*, et al.* Knowledge graph-based recommendation framework identifies drivers of resistance in EGFR mutant non-small cell lung cancer. *Nat Commun* **13**, 1667 (2022).
9. Galli S*, et al.* DNA G-Quadruplex Recognition In Vitro and in Live Cells by a Structure-Specific Nanobody. *J Am Chem Soc* **144**, 23096-23103 (2022).
10. Li C*, et al.* Ligand-induced native G-quadruplex stabilization impairs transcription initiation. *Genome Res* **31**, 1546-1560 (2021).
