# Supplementary material for: Integrative genomic analyses of promoter G-quadruplexes reveal their selective constraint and association with gene activation
Source: Commun Biol. 2023 Jun 10;6:625. doi: 10.1038/s42003-023-05015-6 (PMC10257653; doi:10.1038/s42003-023-05015-6)
Supplement: Supplementary file 3 — Description of Additional Supplementary Files [file 42003_2023_5015_MOESM3_ESM.pdf]

Description of Additional Supplementary Files

File name: Supplementary Data 1
Description: The odds ratios of gene expression difference caused by G-tract variations in different types of promoter pG4s.

File name: Supplementary Data 2
Description: Names of ChIP-seq datasets of histone modification marks, chromatin remodelers and transcription factors, and RNA-seq datasets of HepG2 and K562 cells obtained from the ENCODE database.

File name: Supplementary Data 3
Description: The source data behind the graphs (Fig. 1f-h, 2a-b, 2d-g, 3-4, 5d and 6) in this study.
